# “Meet people where they are”: a qualitative study of community barriers and facilitators to HIV testing and HIV self-testing among African Americans in urban and rural areas in North Carolina

**DOI:** 10.1186/s12889-020-08582-z

**Published:** 2020-04-15

**Authors:** Allison Mathews, Samantha Farley, Donaldson F. Conserve, Kimberly Knight, Alston Le’Marus, Meredith Blumberg, Stuart Rennie, Joseph Tucker

**Affiliations:** 1 Department of Social Medicine, School of Medicine, The University of North Carolina at Chapel Hill, 333 South Columbia Street, MacNider Hall, Room #348 / CB #7240, Chapel Hill, NC 27599-7240 USA; 2 Institute for Global Health and Infectious Disease, School of Medicine, The University of North Carolina at Chapel Hill, 333 South Columbia Street, MacNider Hall, Room #348 / CB #7240, Chapel Hill, NC 27599-7240 USA; 3 Department of Health Policy and Management, Gillings School of Global Public Health, The University of North Carolina at Chapel Hill, Chapel Hill, NC USA; 4 Department of Health Promotion, Education, and Behavior, Arnold School of Public Health, The University of South Carolina, Columbia, SC USA; 5 Department of Clinical Psychology, Fielding Graduate University, Santa Barbara, CA USA; 6 London School of Hygiene & Tropical Medicine, London, UK

**Keywords:** African Americans, HIV, HIV self-testing, Community engagement

## Abstract

**Background:**

HIV testing programs in the United States aim to reach ethnic minority populations who experience high incidence of HIV, yet 40% of African Americans have never been tested for HIV. The objective of this study is to identify community-based strategies to increase testing among African Americans in both urban and rural areas.

**Methods:**

This study conducted focus group discussions (FGDs) informed by community-based participatory research principles to examine African American’s concerns and ideas around HIV testing and HIV self-testing. Participants included highly affected (i.e., PLWH, MSM, PWID, low-income, teens and young adults) populations from African American communities in North Carolina, aged 15 years and older. We digitally transcribed and analyzed qualitative data using MAXQDA and axial coding to identify emergent themes.

**Results:**

Fifty-two men and women between 15 to 60 years old living in urban (n=41) and rural (n=11) areas of North Carolina participated in focus group discussions. HIV testing barriers differed by HIV testing setting: facility-based, community-based, and HIV self-testing. In community-based settings, barriers included confidentiality concerns. In facility-based settings (e.g., clinics), barriers included negative treatment by healthcare workers. With HIV self-testing, barriers included improper use of self-testing kits and lack of post-test support. HIV testing facilitators included partnering with community leaders, decentralizing testing beyond facility-based sites, and protecting confidentiality.

**Conclusions:**

Findings suggest that HIV testing concerns among African Americans vary by HIV testing setting. African Americans may be willing to test for HIV at community events in public locations if client confidentiality is preserved and use HIV self-testing kits in private if post-test social support and services are provided. These community-identified facilitators may improve African American testing rates and uptake of HIV self-testing kits.

## Background

HIV testing programs in the United States aim to reach African Americans, a population which is highly affected by HIV. African Americans account for the majority of new infections nationally, yet only 40% have ever tested for HIV [1]. In North Carolina, African Americans make up 22.1% of the population and 57.1% of new HIV infections among 15 to 34 year olds [2]. It is important to identify strategies to address the unique challenges that face African Americans in both urban and rural settings.

Several barriers are known to impact testing among African Americans, including HIV-related stigma [3, 4], reduced access to healthcare [5], low HIV knowledge [3], low perception of risk [3, 6–8], and fear of a positive result [8]. A major difficulty in reaching African Americans for testing continues to be the overlapping social stigmas attached to being a racial/ethnic minority, low-income, and/or labeled as promiscuous [5]. While these trends have been noted among African Americans living in urban areas, there is less data among African Americans living in rural areas, who make up 8.4% of all rural residents in the United States and 20.4% of rural residents in North Carolina [9–11]. Rural residents are often diagnosed later than urban residents [12]. The delay in HIV testing among rural residents is due to fewer resources for HIV testing and providers who treat people living with HIV; greater distances between residents and services; and stigma [13]. There is a need to identify new, community-driven ways to engage African Americans in testing that reflect the experiences of underserved, predominantly African American communities in urban and rural areas. This study uses community-based participatory research principles to guide an examination of African Americans’ barriers and facilitators to HIV testing and HIV self-testing (henceforth, HIVST) in urban and rural areas in North Carolina.

## METHODS

### Setting

The study was conducted in an urban city and two rural towns in North Carolina. These geographic locations were chosen because they are in counties with a higher HIV prevalence compared to other counties [2]. Durham County, the urban area chosen for this study, has a three-year average HIV infection rate of 26.8 per 100,000 population (2015-2017) [2]. The two rural counties chosen for this study, Edgecombe (29.2 per 100,000 population HIV infection rate) and Wilson (15.2 per 100,000 population HIV infection rate), are adjacent to each other and have the 2nd and 19th highest 3 year average (2015-2017) HIV infection rates, respectively, in the state [2].

### Participant Eligibility

Eligible participants included African Americans aged 15 years and older. While the eligibility criteria for participation was general, we targeted recruitment to reach a cross section of individuals from highly affected populations (i.e., People living with HIV (PLWH), men who have sex with men (MSM), people who inject drugs (PWID), low-income, teens and young adults) most affected by HIV in North Carolina. We included PLWH, adults older than 35 years old, and those who had been tested previously because they could provide insight into their previous testing experiences, their experiences being diagnosed as positive, and how people who are 15 to 35 years old in their community might feel when getting tested.

### Recruitment

Our recruitment activities included sending flyers, emails, social media advertisements, and making phone calls to community-based organizations and local businesses. We also recruited at Historically Black Colleges, organizations serving people who use drugs, low-income housing developments, African American teens, and networks of men who have sex with men. We did not ask people to disclose their HIV status; however, we recruited from organizations known to serve PLWH. Some participants voluntarily revealed their status during focus group discussions. It is important to note that participants voluntarily disclosed their status in focus group discussions and others participating in this study may have been living with HIV but did not volunteer their status during discussion. We obtained ethical approval from the Institutional Review Board at the University of North Carolina at Chapel Hill. Informed consent was obtained from all participants and written consent and adolescent assent were provided by all participants. Those aged 15 to 17 years old provided assent and had parental/guardian consent to participate in the focus group discussions.

### Data Collection

We used community-based participatory research principles to seek community feedback on the best ways to promote HIV testing among African Americans in urban and rural areas of North Carolina [14]. We conducted nine focus group discussions, two of which were in rural areas, each lasting approximately 1.5 to 2 hours. The focus group guide was adapted from a study by Mathews et al. 2018, which was designed to elicit community-based ideas on the best ways to engage the public about HIV cure clinical research (See Additional file 1 for focus group guide) [15]. We divided the focus group discussions so that people from highly affected populations (i.e., PLWH, MSM, PWID, low-income, teens and young adults), rural and urban, and similar peer networks could participate in the same sessions. The sessions assessed community-level barriers and facilitators to HIV testing and HIVST among African Americans in general, then we asked participants to generate ideas for ways to promote testing among Black young adults aged 15 to 35 years old because they are at the highest risk for acquiring HIV in North Carolina [2]. Participants received $20 gift cards for their time. Informed consent procedures were undertaken, and consenting participants were asked to remain in the room to answer demographic questions and participate in the focus group. To protect participants’ identities, we refer to their locations as urban and rural areas rather than identifying the specific area where they live, removed references to gender, and used age ranges rather than specific ages.

### Measures

Focus group questions were adapted from items used in a previous HIV qualitative research study conducted in the same state [15]. Questions asked participants to reflect on how to reach Black community members to promote HIV testing in their areas. To assess community motivations to test for HIV, a sample item included, “What would it take to get people tested for HIV in this area?” To assess HIV testing willingness in different locations, sample items included, “What would motivate people to get tested at events?” To assess willingness to use HIVST kits, sample items included, “How willing do you think people would be to administer an at-home testing kit?” and “How might HIVST help remove some barriers [to HIV testing]?”

### Data Analysis

Digital transcriptions of qualitative data were analyzed using a qualitative data analysis software called MAXQDA. Two individuals independently read transcripts to identify deductive codes and developed a codebook. We discussed discrepancies in coding to reconcile differences and collaboratively finalized the codebook. Deductive codes were developed based on the semi-structured focus group guide (see Additional file 1), and emergent themes were identified during subsequent transcript readings. To identify emergent themes, we analyzed blocks of text to elucidate descriptive and interpretive meaning using axial coding and memo writing.(15) We categorized the types of HIV testing settings based on types defined by the WHO: 1) community-based (e.g., community events, concerts), 2) facility-based (e.g., HIV testing clinics, community health centers, testing kiosks), and 3) HIVST (see Table 1) [16].

**Table 1:** Barriers and Facilitators to HIV Testing in Rural and Urban Areas of North Carolina by Testing Settings

Barriers	Facilitators
**Community-Based Testing***Health fairs, community events, etc.*	
• People may be uninterested in attending an event that was specifically about HIV	• Host events on the weekends in places that are easily accessible to the public, like college campuses, health fairs, or Walgreens
• HIV test providers might not return to maintain long term relationships with community beyond testing event	• Provide testing at popular venues or events with food, entertainment, and community leaders
• Some people may not want to be in crowds	• Partner with local organizations like barbershops, dance classes, speed dating events to offer testing
	• Enlist community leaders (church leaders, etc.)
**Facility-Based Testing** *Clinics or health departments*	
• Clinic-related stigma (stigma connected to the physical building being associated with HIV and the potential for a positive result)	• Setting up private spaces in public spaces (private testing cubicles within health departments, social services, CVS, etc.)
• Stigma exacerbated by clinic employees	• Hiring health department vans to attend public events
**HIV Self-testing (HIVST)** *At home, self-testing kits*	
• Inability to understand HIVST kits	• Watch instructional video at home
• Lack of attention to instructions or long video included with HIVST kit	• Create more engaging, shortened instructional videos
• Potential breaches in confidentiality (specifically for people aged 15-17)	• Call insurance, receive individual insurance card
• Lack of social support	• Have someone you trust with you as you administer the test

## Results

We hosted nine focus groups with 52 African American participants (n=31 men; n=21 women; ranging in age from 15 to 60 years old) from February to June 2017. Participants lived in urban (n=41) and rural (n=11) areas and included several highly affected populations, such as women living in public housing (n=5), heterosexual Black men (n=13), men who have sex with men (n=5), people who use drugs (n=3), and people who voluntarily identified as living with HIV (n=9). Table 2 provides a full demographic description of participants. Some participants voluntarily disclosed they were living with HIV during focus group discussions. Emergent themes reflected concerns about HIV testing stigma in different testing settings (i.e., community-based, facility-based, and HIVST) and suggestions on ways to address those concerns through the use of public opinion leaders, community events, and protection of confidential information.

**Table 2:** Focus Group Participant Demographics (N=54)

	Rural (n=10)	Urban (n=44)
**Age**		
15-17 YO	1	8
18-23 YO	1	14
24-29 YO	1	9
30-35 YO	2	8
36-41 YO	0	3
42-52 YO	0	0
48-53 YO	3	1
54+ YO	2	1
**Sex/Gender**		
Male	7	28
Female	3	16

### HIV Testing and HIV Self-Testing Barriers

To better understand HIV testing among African Americans, we assessed barriers and facilitators to HIV testing. HIV testing barriers included fear of confidentiality breaches, discriminatory treatment by healthcare professionals, lack of consistent community presence by HIV test providers, stigmatization from religious leaders, lack of information about post-test options, and fear of improper use of HIVST kits (see Table 1). Many of these barriers were related to HIV test-associated stigma.

#### Young people’s fears of confidentiality breaches

Younger participants, including teens and college students, (n=10) explained that they had a concern about potential breaches of their confidentiality while being tested for HIV. A teenager from rural North Carolina mentioned that in such a small community “[people] don’t want to go to the clinic because they know somebody there.” The teen went on to explain that most community members do not know about privacy rules, and that it always “goes back to stigma, fear of people finding out.” Some youth participants (n=4) expressed concern about breaches of confidentiality because of the possibility that, as one teenager from urban North Carolina explained, “parents at home [could] find out.” When we asked teen participants about the possibility of using HIVST kits at home, they also expressed concerns about confidentiality. In particular, concerns about confidentiality were related to the stigma of buying HIVST kits in public and using their parents’ insurance to purchase the kits. A teenager from an urban area explained: “Unless you’re buying it online and it’s discreet [I wouldn’t use a HIVST kit.] Who wants to go to Walgreens and get a test?” Another teenage participant from an urban area added: “Sometimes your insurance has to take care of certain things. [I would be] like, ‘Ma, I need the insurance card.’ ‘What for?’ That would be a thing.”

#### Stigmatizing treatment by healthcare professionals

Stigmatizing treatment by healthcare professionals while getting tested was a barrier some (n=4) participants in urban areas identified as a deterrent for community members. An adult participant living in urban public housing described frustration with health workers: “[They treat you] like you’re [not] a person, not trying to build a relationship and just doing their job. It makes people feel uncomfortable.” Participants relayed experiences with poor treatment by clinic employees. A participant who self-identified as living with HIV (in their 50’s, urban area) described their experience getting tested at an urban health department in North Carolina: “I contracted HIV from my husband who did not tell me his status. I had to go in the side entrance of the health department and the stigma was so high I didn’t want people to know. It was a lot of shaming in that clinic and when I went back to get my results the man told me I was HIV-positive.” When this participant was diagnosed with HIV, the clinic employee seemed to encourage self-stigmatization, telling them “you’re not upset. What’s wrong with you?”

#### Stigmatization from religious leaders

Many participants (n=16), from both urban and rural areas, explained the stigma connected to HIV testing was influenced by community and faith-based leaders. A participant who self-identified as living with HIV (in their 50’s, urban area) made the point that some faith-based leaders refuse to discuss HIV: “The faith-based organizations talk about cancer or other topics but they will not dare to focus on that topic. They will talk about fear, alcohol, or even sex but they won’t talk about HIV. No matter what faith they are they should address this. People need to face reality that people are having sex whether married or single. Don’t wait for the health department to do a health fair. It’s your [the faith leader’s] job to talk about it.” In this example, there was no explicit stigma by the religious leader, but implicit stigma in the silence around the topic.

#### Fear of results

The lack of information about how to deal with an HIV test result created anxiety for some (n=8). “The anxiety about the results is stressful”, said a participant (in their 30’s, urban area). Supporting the previous participant’s statement, another participant (in their 30’s, urban area) stated, “Like [they] said it is the stress of waiting.” Participants explained that part of the anxiety associated with HIV testing was due to a fear of the unknown regarding test results. A participant (in their 20’s, urban area) said: “Getting tested is scary cause you don’t know what you’re getting into. It’s hard out here by yourself. To be in the company of another person and feel their passion, but to think that you would catch something, that’s like…It’s like going on a ride and losing control.”

A barrier some participants associated with HIV testing was the potential for death and social isolation from being diagnosed as HIV positive. One participant (in their 30’s, rural area) explained how some community members avoided testing because of a fatalistic attitude about a positive HIV result: “It’s depressing. People gotta die of something, so I would rather not know and just die.” The same participant expressed frustration with not knowing how to convince people to test for HIV: “We were trying to tell him ‘You don’t have to die from HIV. You can live.’ He was just like ‘nah it’s a death sentence. I don’t want to hear it. I don’t want to get any care for it.’ How do you respond to that?”

#### Challenges with HIVST kits at home

Some participants (n=6) preferred the option of testing in the privacy of one’s home and suggested using HIVST kits at home as a viable alternative to getting tested in public spaces. A participant (in their 30’s, urban area) suggested, “The opposite of an event is having something more private. It doesn’t require someone to come out somewhere and [they can] watch something from their home in their own time. People don’t like being in crowds, especially if it's not something that they’re passionate about.” However, a participant (in their 20’s, urban area) expressed concerns about not having support to deal with a result after using a HIVST kit: “After you’ve done the test, where do you go to get help? With the test, is there any sort of [help?]” The moderator assured participants that HIVST kits come with hotlines for post-test counseling and instructions to properly use the kit. However, another participant (in their 30’s, urban area) remained critical: “I never read instructions. That’s a problem especially with something so sensitive. That’s a challenge people aren’t going to read the instructions.” The lack of knowledge about how to use HIVST kits, coupled with fear of breaches of confidentiality and lack of social support, were associated with negative perceptions about testing in private locations like homes. However, participants were not entirely opposed to using HIVST kits in private spaces; they just wanted assurance that risks were minimized through access to resources like hotlines and easy-to-use instructions.

### HIV Testing Facilitators

**Fig. 1:**
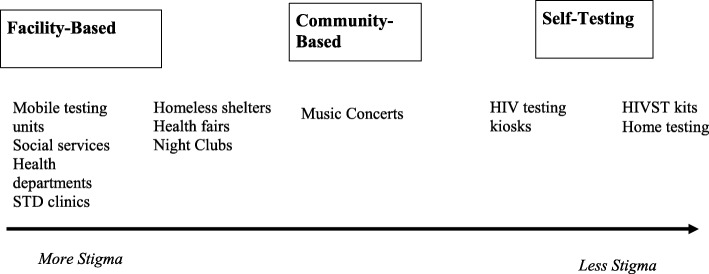
Experiences with HIV-related stigma in relation to HIV testing settings

Participants identified many HIV testing facilitators among African Americans. The facilitators were often dependent on the location of testing. We developed a continuum of participants’ experiences with HIV-related stigma based on the way participants described stigma attached to different types of HIV testing settings (See Fig. 1). Participants’ experiences with HIV-related stigma seemed to peak at semi-public locations and dissipate outward on the privacy spectrum in both directions. Figure 1 demonstrates where examples of different HIV testing location types may fall on the privacy spectrum. Of note, the participants’ experiences with HIV-related stigma do not follow the same direction as the privacy levels of testing location types.

### Facilitating HIV Testing in Community Settings

The majority of participants (n=22) in both urban and rural areas preferred HIV testing in community-based settings. We define community-based settings as public spaces where anyone can enter or leave, such as health fairs, community events, and nightclubs. There is little to no expectation of privacy in these spaces. Participants emphasized the potential of public events as a way to reduce stigma associated with HIV testing. Three of the most popular suggestions included: 1) promoting testing at community-based sites; 2) partnering with community-based organizations (CBOs); and 3) enlisting popular opinion leaders to encourage HIV testing.

#### Promote Testing at Community-Based Sites and Events

In urban areas, participants emphasized the benefits of community-based sites as a way to encourage people to test for HIV and “meet people where they are.” A participant (in their 50’s, urban area) stressed the importance of “meet[ing] people at their comfort level. The goal is to prevent, make aware, educate…meet at their home, in a small group setting, non-confidential. It may help them make leaps and bounds to get them where they need to be.” In particular, participants discussed how health fairs, schools, churches and concerts would be beneficial because they would increase HIV awareness and make testing more accessible to the general public. For example, a participant (in their 30’s, urban area) explained: “Events on the weekends or evenings that are readily available to them [the public]. Some people [have] their stigmas about government-provided services. Take advantage of the services given to you. [It’s better to go to] college campuses, health fairs, [and] Walgreens [to get] the flu shot so you don’t have to go anywhere [far] and can do it on your lunch break.”

In both urban and rural areas, participants identified certain locations that could attract different types of people to get HIV tested. A self-identified gay participant (in their 30’s, rural area) explained, “I know downtown is the spot for everybody. So, when you’re hosting an event it would be downtown. I mean really that’s where everybody goes. Straight, gay, whatever.” Similarly, another participant (in their 30’s, urban area) mentioned events such as “chili cook-offs [and] Black hair product expos” and locations such as “barbershops [or] small businesses” as prime opportunities for HIV testing. Importantly, the public events that participants suggested were not specific to HIV, but were convenient events and locations that offered flexibility for people to test in low-pressure environments. It was also important to a maintain consistent presence in community-based settings. A participant living in urban public housing described their frustration: “[We need] a follow up. If you come out and test somebody, [you need] to come back, not necessarily to see if they’re positive, but to check in on them.” They criticized test providers for being inconsistent and not taking time to develop meaningful relationships with community members.

#### Partnering with Community-Based Organizations

For both urban and rural participants, it important to connect testing to public sites and events and connect with local, trusted CBOs and businesses to promote the events. For example, a participant (in their 50’s, urban area) suggested that CBOs like “fraternities [and] church organizations” were well respected in the community and especially influential to Black men. A participant (in their 30’s, urban area) offered another suggestion: “get with the leaders of different groups that people are associated with and have free drives.” Offering testing at community-based events was also mentioned as a way to mitigate HIV testing-related stigma. In particular, a participant (in their 30’s, urban area) said: “Don’t reinvent the wheel. Tack it onto other events. And that way you are normalizing testing instead of making it a big deal.” The suggestions reflect the idea that having events in partnerships with CBOs may help engage African Americans in HIV testing.

#### Enlisting Community Leaders for Public Events

In addition to partnering with CBOs and businesses, participants suggested partnering with community leaders, such as faith-based leaders and celebrities, to promote HIV testing. In rural areas, faith-based leaders seemed to hold particular importance. For example, a participant (in their 20’s, rural area) suggested that faith leaders should serve as role models for others: “My first time [getting tested] was at my church through a health fair. My pastor was the first person to get tested and it removed the stigma. Everyone followed after that.” Given the influence that faith-based leaders have on community members’ decision-making around their health care and health behaviors, participants emphasized the importance of engaging them in discussions about HIV in addition to other health topics like cancer.

In addition to faith leaders, participants suggested partnering with musicians at concerts to promote HIV testing. In the rural areas, participants suggested faith-based music while in urban areas participants provided suggestions from hip hop and popular culture. For example, a participant (in their 50’s, rural area) said that the area was known for gospel music and it attracted large crowds: “[This] used to be a concert area for gospel singing. This is where most of the stigma is and surrounding areas in the churches. I see pastors in the pulpits shining their lights on the congregation. A concert would really draw people and have speakers.” In contrast, another participant (in their 20’s, urban area) suggested: “Have someone prominent to speak on why it’s so important to get tested. We normally have people to get tested, but never have people to say why it was important. If [a famous hip hop video DJ] came to say why it was important, then we would pay attention.” Similarly, a participant (in their 20’s, urban area) suggested: “Have a benefit, an event that’s centered around a campaign. Have a well-known person, fair, or concert to explain why it’s important to get tested. If there’s just a brochure, I won’t pay attention.” Importantly, the idea of prominent media personalities or entertainers participating in testing events and sharing their experiences was a popular suggestion for participants from both urban and rural areas to help attract people to testing events and bring awareness to larger communities who may be unaware of local needs.

### Promoting HIV Testing in Facility-Based Settings

We categorized locations such as health departments and STD clinics as facility-based settings because they are associated with specific buildings and provide private, enclosed spaces to discuss HIV testing and care. Patients may enter these spaces with the expectation of privacy, one-on-one conversations, and on-site counseling when getting tested for HIV. Some participants (n=7) listed these locations as the most common places to get tested. Indeed, a participant (in their 20’s, urban area) stated that testing for HIV is easy “inside of clinics, inside of social services, anywhere that you see that civil people are they do it.” However, facility-based settings were not the most preferred places to receive an HIV test, particularly in rural areas, because of concerns with stigmatizing experiences with healthcare workers, such as being labeled as promiscuous or assumed to be HIV positive.

Participants offered potential opportunities for improving HIV testing within semi-public spaces. One participant (in their 20’s, rural area) noted that self-service kiosks could make facility-based spaces more appealing, comparing HIV testing to mental health assessments. They stated, “At the health department, there’s a computer station for mental health. They can sit in a private space, [a] cubicle and take a survey and assess their mental health and give them a resource on where they can go. Yes, a lot of people use it.” It is possible that associating the testing kiosk with other health conditions may make it an appealing option for those accessing facility-based locations for HIV testing [17–19]. Another example of an alternative facility-based setting was a health department sponsored van for testing at public events. The same participant mentioned a time when a health department van came and gave free, confidential testing. People were motivated to be tested in the van because their pastor went first, “remov[ing] the stigma. Everyone followed after that.” The testing van was an opportunity to provide testing at a public event while still having the privacy of a clinic. However, rural participants cautioned against relying on vans solely for the purpose of STD/HIV testing because they would be stigmatized as spaces associated with STDs and HIV.

### Facilitating HIV self-testing

Focus group participants offered some suggestions to improve experiences with at-home testing and the use of HIVST kits in private locations. In particular, teen participants were primarily focused on identifying ways to protect confidentiality and circumvent inadvertent disclosure to their parents/guardians. One teen participant (urban area) suggested for other teens to “get [their] own insurance card.” Similarly, other focus group participants identified ways to change attitudes around testing at home to encourage people to pay attention to the instructions. For example, a participant (in their 20’s, urban area) stated, “My perspective is: your health is at stake and the health of others around you. It would behoove you to take a few minutes to read the instructions just this one time. This is your life and your health.” To encourage people to read the instructions and better comprehend proper HIVST kit use, another participant (in their 30’s, urban area) suggested to “train the pharmacist to use the test. Maybe the person could stick around or they won’t. It might help bridge the gap and be more effective. I check my heart rate. I get physicals.”

## Discussion

To better understand African Americans’ perspectives on how to improve HIV testing experiences in both urban and rural areas, we conducted focus group discussions with highly affected populations in North Carolina. Our analysis reflects a combination of concerns of African Americans in both urban and rural locations. Urban and rural participants described similar barriers to HIV testing, including concerns about potential breaches of confidentiality, negative treatment by healthcare professionals, lack of a consistent community presence from HIV test providers, stigmatization from religious leaders, lack of information about post-test options, and fear of improper use of HIVST kits at home. Despite these barriers, participants noted several facilitators to normalize HIV testing among African Americans and reduce HIV-related stigma, including partnering with CBOs and community leaders to promote public testing events, setting up HIV testing kiosks in convenient locations, providing informational and social support to people using HIVST kits, and protecting confidentiality.

Community settings may be more effective sites for promoting HIV testing, identifying new positives, and for reaching African Americans than STD clinics [1, 20]. Our study findings revealed that African American community members in both rural and urban areas prefer public events, such as concerts, health fairs and nightclubs as HIV testing sites. Other studies have shown success in implementing community-level interventions to increase testing among African Americans, particularly on HBCU campuses and surrounding communities [21], at gay pride events [22], and at pharmacies and retail clinics [23]. While testing at public events was the most popular suggestion, participants expressed some potential challenges. Barriers included concerns about confidentiality and the inconsistency of some HIV test providers who may not maintain a sustainable presence as a resource to community members. Given the severe lack of public events that offer testing in rural areas, it may be important to leverage current public, community-based spaces, events and leaders to normalize testing experiences and reduce HIV-related stigma.

HIV-related discrimination and poor treatment in healthcare settings was identified as another barrier to HIV testing. This finding is consistent with US research showing pervasive HIV-related discrimination in clinical settings [8, 24, 25]. Currently, the majority of HIV testing in the United States occurs in STI clinics, which are more successful at identifying new positives among White men and those already connected to healthcare [20]. Participants’ preference for decentralized, non-clinical community-based sites for HIV testing were related to their experiences with HIV-related stigma, perpetuated by healthcare workers, as well as the social stigma attached to the clinical testing sites. These preferences may have been influenced by the fact that participants included those who had been tested previously for HIV and/or were HIV-positive. HIV-related stigma among healthcare workers has been shown to be pervasive in the Deep South, particularly among those who are Protestant, White, and working in an HIV/STI clinic [8]. As a consequence, highly affected populations within African American communities delay HIV testing due to anticipated HIV stigma [8], and regard local health departments, physical offices and drug use treatment centers as places to avoid being tested [25]. Participants’ suggestions to disconnect testing from clinical settings and limit interactions with health care providers (i.e., testing kiosks, HIVST kits, community testing) may assist with reducing negative perceptions about HIV testing experiences. While non-clinical testing may help reduce stigma, it is also important to identify ways to improve linkage to care for people tested in community settings [26, 27]. Faith-based and community-based leaders could not only help change norms about HIV as a taboo topic, but also serve as an ongoing local voice for testing promotion and awareness, filling in the gaps between larger public events like health fairs that promote HIV testing.

In an effort to find alternatives to testing in community-based and facility-based settings, participants suggested that home testing and HIVST kits might be useful for those concerned about stigma and potential breaches of confidentiality. Participants identified several barriers to using HIVST kits, including fear of breaches in confidentiality, stigma, fear of social rejection, and lack of information about how to properly use the HIVST kit. Younger people were concerned about adults finding HIVST kits or having access to their health information from insurance records. These concerns reflected a vulnerability to breaches in confidentiality among participants who may still be dependent on parental and guardian support, which can extend to 26 years of age under current health insurance coverage policies in the United States. In contrast, adult participants were interested in home testing because it appealed to them to be able to test in the comfort of their home. Similarly, a previous study of young Black men who have sex with men in North Carolina found that the convenience of HIVST kits was more appealing to those with sufficient monthly income and high education levels as compared to those with low income and low education levels [28, 29]. Lastly, concerns about the proper use of HIVST kits have been shown in previous studies among key populations who were unsure about their ability to afford HIVST kits and properly use them [28, 30]. Studies have demonstrated that people are generally able to properly use HIVST kits [31]; however, there may be a need to improve the dissemination of messages assuring people that there are resources available to educate and support HIVST kit users.

There were some limitations to this study. The small sample size (n=52) limits generalizability of the findings among African Americans in the southeastern United States. Although we had fewer participants from rural areas as compared to urban areas, a strength of this project was the inclusion of rural as well as urban perspectives, especially given the pervasiveness of HIV-related stigma in rural areas. Additionally, many participants were people living with HIV and/or members of community-based organizations and college student populations, which may have biased their suggestions for promoting public events as facilitators of HIV testing. However, the sample included highly affected subgroups, such as low-income women who have sex with men, men who have sex with men, people who used injecting drugs, and teens, within the African American community from both rural and urban areas. These subgroups provided a diverse set of perspectives on HIV testing and HIVST experiences such that we gained a better understanding of thoughts from people who had never been tested, recollections about prior testing experiences, a sense about African American community perceptions about HIV testing in urban and rural areas of North Carolina, and experiences after receiving a positive result.

## Conclusion

Our study has implications for HIV testing among African Americans in urban and rural areas of the American South. For public health practitioners and testers, it may be important to identify ways to decentralize HIV testing by promoting public testing events connected with organizations already serving groups that need HIV testing, particularly in rural areas. Further, there should be expanded resources for conducting testing in non-clinical spaces to assist with normalizing HIV testing. Additionally, there is a need to educate the public on how to use HIVST kits while providing access to social support and protecting confidentiality, particularly for teens and those living in rural areas. Gauging community-driven suggestions for conducting HIV testing is necessary to implement effective screenings in diverse communities. Our findings demonstrate that community-based participatory research principles, when used to identify ways to improve testing among African Americans in urban and rural areas of North Carolina, can yield practical suggestions to reduce stigma associated with HIV testing and HIVST.

## Supplementary information

**Additional file 1.**

## Data Availability

Data sharing is not applicable to this article as no quantitative datasets were generated or analyzed during the current study. The data included in this study are qualitative and sensitive in nature, potentially compromising participant confidentiality. It is possible to obtain an anonymized data set from the corresponding author on reasonable request.

## Abbreviations

CBO: Community based organizations
CBPR: Community-based participatory research
HIV: Human immunodeficiency virus
HIVST: Human immunodeficiency virus self-testing
MSM: Men who have sex with men
PLWH: People living with HIV
STD: Sexually transmitted diseases
STI: Sexually transmitted infections
