# Supplementary material for: “Meet people where they are”: a qualitative study of community barriers and facilitators to HIV testing and HIV self-testing among African Americans in urban and rural areas in North Carolina
Source: BMC Public Health. 2020 Apr 15;20:494. doi: 10.1186/s12889-020-08582-z (PMC7161271; doi:10.1186/s12889-020-08582-z)
Supplement: Supplementary file 1 — Additional file 1. [file 12889_2020_8582_MOESM1_ESM.docx]

APPENDIX I

| **MODERATOR:** We would like to talk about how to involve Black youth and young adults in *name of city* between 15 and 35 years old in a campaign that promotes HIV testing and the use of HIV self-testing kits in the *name of city* community. We want to know what elements (e.g., types of recruitment events, focus topic for campaign, format of campaign, etc.) we should use to promote HIV testing and the use of self-testing kits among Black youth and young adults in your community. | |
| --- | --- |
| **QUESTIONS:** | **PROBES:** |
| 1. Can you tell me about the venues that Black young adults in *name of city* attend? | What types of venues are these?  Church, club, bar?  Can you describe the crowd?  Male, female, mixed?  Gay, straight, mixed?  Students, young professionals, locals?  Hip hop?  Age range? |
| 2. What are some examples of events that are really successful at getting Black young adults to attend? | What do these events focus on? |
| 3. What is it about these events or activities that draw Black young adults? | What is the environment/scene like at these events?  Which sponsors attract the most people to these events/activities?  Are any of these led by Black young adults?  Examples of unsuccessful events?  Can you give me some reasons why it was unsuccessful? |
| Ok, so now let’s talk about events that might focus on issues in the Black *name of city* community.  4. What events for Black young adults are held in *name of city* that address issues in the Black community? | What do these events focus on?  What about HIV/AIDS?  What is the age range for the people that attend these events?  What do people do at these events?  Are these events popular/highly attended? |
| 5. What types of activities and events addressing HIV/AIDS would Black young adults in *name of city* be willing to participate in? | What would it take to get you there and KEEP you there?  How can we make events fun & get across HIV/AIDS info at the same time?  Who would be most likely to attend?  Female, male?  Gay, straight, mixed?  Students, young professionals, locals? |
| 6. Which groups or organizations should we partner with to hold these events for Black young adults? |  |
| 7. What's the best way to get the word out about events if we want to reach Black young adults? | Which radio stations?  Are there any print media that would reach Black young adults?  Facebook?  Text Messages?  Twitter?  Instagram?  SnapChat?  TV stations?  Celebrities? |
| 8. What would prevent Black young adults from attending these events? | Would cost be a factor?  Would a free event increase attendance?  Would days of the week, time of the year, times of the day, location have an influence? |
| We are trying to bring community members together to design a campaign to promote HIV testing.  9. What are some of the biggest barriers to HIV testing in *name of city* for Black young adults? | What else could we do besides events to involve people in this project? |
| 10. What are some of the biggest motivators for Black young adults to get tested for HIV? |  |
| Now we want you to break into groups and brainstorm ways to promote HIV testing among youth and young adults between 15 to 35 years old in your communities. In your groups, we want you to think about the barriers and motivators we discussed and think about a catchy campaign or contest idea that could motivate Black young adults to get tested for HIV. | *Focus group participants break up into groups of 3 to 4 and brainstorm ideas for 10 minutes. At the end of the 10 minutes, participants report back their ideas on ways to promote HIV testing among Black youth and young adults in their communities. |
| Thank you for sharing your ideas about ways to raise awareness about regular HIV testing. I would like to now ask you a few questions about how to raise awareness about HIV self-testing for young Black adults. | HIV self-testing is a way of seeing for yourself if you have contracted HIV, instead of having to see a doctor and ask for a test. Special kits let you swab your gums to collect some saliva, and then test the saliva to see if there’s any sign of HIV in your body. You can do this entirely by yourself in the privacy of your own home, and it takes about 20-30 minutes to complete. You can buy kits at pharmacies and drug stores, and they cost around $40. You can also ask for an oral HIV test at the clinic instead of a blood test. |
| Were you familiar with HIV self-testing before today? |  |
| Earlier you mentioned a few barriers to HIV testing for Black young adults in *name of city*.  How might HIV self-testing help remove some of these barriers? |  |
| What do you think would be some barriers to HIV self-testing for Black young adults in *name of city*? |  |
| How willing do you think people would be to administer an at-home testing kit? |  |
| If you could design a contest about raising awareness for HIV self-testing, what would it look like? |  |
